# Transnasal Penetrating Intracranial Injury with a Retained Foreign Body: Diagnostic Challenges and Endoscopic Surgical Management—A Case Report

**DOI:** 10.3390/life16081321

**Published:** 2026-08-12

**Authors:** Stoyan Markov

**Affiliations:** 1 Department of Otorhinolaryngology, Faculty of Medicine, Medical University of Plovdiv, 4002 Plovdiv, Bulgaria; stoyan_bgus2002@yahoo.com; 2 Department of Otorhinolaryngology, St George University Hospital, 4001 Plovdiv, Bulgaria

**Keywords:** transnasal penetrating injury, intracranial foreign body, endoscopic endonasal surgery, cerebrospinal fluid leak, suicide attempt

## Abstract

**Introduction:** Transnasal penetrating intracranial injuries represent an exceptionally rare form of traumatic brain injury, characterized by high mortality and a substantial risk of severe neurosurgical and infectious complications. Self-inflicted injuries involving the penetration of a foreign body through the nasal cavity into the brain are exceedingly uncommon and require prompt diagnosis and a multidisciplinary treatment approach. **Objective:** This study aimed to present a rare clinical case of transnasal penetration of a foreign body (a ballpoint pen refill) into the brain parenchyma during a suicide attempt, and to describe the diagnostic and therapeutic approach. **Case Report:** A 46-year-old woman with an acute psychotic disorder was admitted following a suicide attempt involving the insertion of a ballpoint pen refill through the right nasal cavity. Computed tomography revealed a retained intracranial foreign body embedded within the brain parenchyma, traversing the cribriform plate and associated with pneumocephalus. The foreign body was successfully removed via an endoscopic endonasal approach. Owing to the cerebrospinal fluid leak that developed after removal of the foreign body, reconstruction of the anterior skull base defect was performed using fascia lata and tissue adhesive. The postoperative course was uneventful, with no neurological complications and gradual resolution of the intracerebral hemorrhagic changes and pneumocephalus. The patient was discharged without neurological deficits and was referred for psychiatric treatment with a diagnosis of catatonia accompanied by episodes of psychomotor agitation. **Conclusions:** Transnasal penetrating brain injuries are rare but potentially fatal. Early radiological diagnosis, meticulous preoperative planning, and an endoscopic endonasal approach enable safe removal of the foreign body and reliable reconstruction of the anterior skull base defect. Multidisciplinary collaboration among neurosurgeons, otorhinolaryngologists, radiologists, and psychiatrists is essential for achieving favorable outcomes in these patients.

## 1. Introduction

Penetrating injuries to the brain caused by foreign bodies traversing the skull are rare but potentially life-threatening conditions, posing significant diagnostic and therapeutic challenges for clinicians. Among these, transnasal intracranial injuries—defined as the penetration of a foreign body into the brain through the nasal cavity—represent a particularly uncommon entity because of their exceptional rarity, atypical clinical presentation, and high risk of severe complications [1,2,3].

Penetrating cranial injuries are defined as injuries caused by a low-velocity object with a small impact surface area [1]. The transnasal route of intracranial foreign body penetration closely resembles the surgical approach used in transsphenoidal pituitary surgery, passing through the nasal cavity, sphenoid sinus, and skull base. This anatomical pathway explains how even common everyday objects may reach critical intracranial structures [1,4]. Reported cases have involved a wide variety of foreign bodies, including chopsticks [2,5], wooden branches and sticks [3,6], pencils [6], pens [7], needles [8], and umbrella tips [1], each presenting unique diagnostic and therapeutic challenges.

A substantial proportion of the published cases involve children who sustained these injuries following accidental falls onto sharp objects [1,3]. Although considerably less common, transnasal penetrating injuries have also been reported in adults, most often in the context of deliberate self-harm or suicide attempts [2,7]. Penetrating injuries of the midface sustained during suicide attempts pose significant clinical challenges, with reported mortality rates reaching 50% in published case series [1,3]. Moreover, many patients present to the emergency department without a reliable history, without obvious external signs of injury, and in the absence of the penetrating object responsible for the trauma [1,3,4].

Imaging plays a pivotal role in the diagnosis and assessment of the severity of these injuries. Conventional radiography has limited sensitivity, particularly for non-metallic foreign bodies. Wooden foreign bodies exhibit radiodensity similar to that of soft tissues and air within the paranasal sinuses, making them difficult to detect on plain radiographs [3,5,6]. Computed tomography (CT) is the imaging modality of choice for determining the trajectory of penetration, as well as the size and precise intracranial location of the retained foreign body. Assessment of vascular injury is performed using CT angiography, conventional angiography or magnetic resonance imaging (MRI). For most surgeons, MRI is the preferred imaging modality for preoperative evaluation and surgical planning [3,4,6,9]. Retained intracranial wooden foreign bodies are particularly hazardous because of their porous organic structure, which predisposes them to bacterial colonization and the subsequent development of brain abscesses, meningitis, and other infectious complications, sometimes occurring years after the initial injury [5,6].

From a psychiatric perspective, suicide attempts involving penetrating injuries constitute a small but clinically significant subgroup. A systematic review of the literature has shown that self-inflicted penetrating injuries occur predominantly in men and are most commonly associated with mood disorders and schizophrenia spectrum disorders [7]. The inadequate and inconsistent reporting of the psychiatric characteristics of patients who attempt suicide by means of penetrating cranial injuries in the published literature limits our understanding of this unique patient population and underscores the need for a multidisciplinary approach to their management [7].

**The aim** of the present study is to report a rare case of transnasal intracranial penetration of a foreign body (a ballpoint pen) into the brain during a suicide attempt.

## 2. Case Presentation

**Figure 1:**
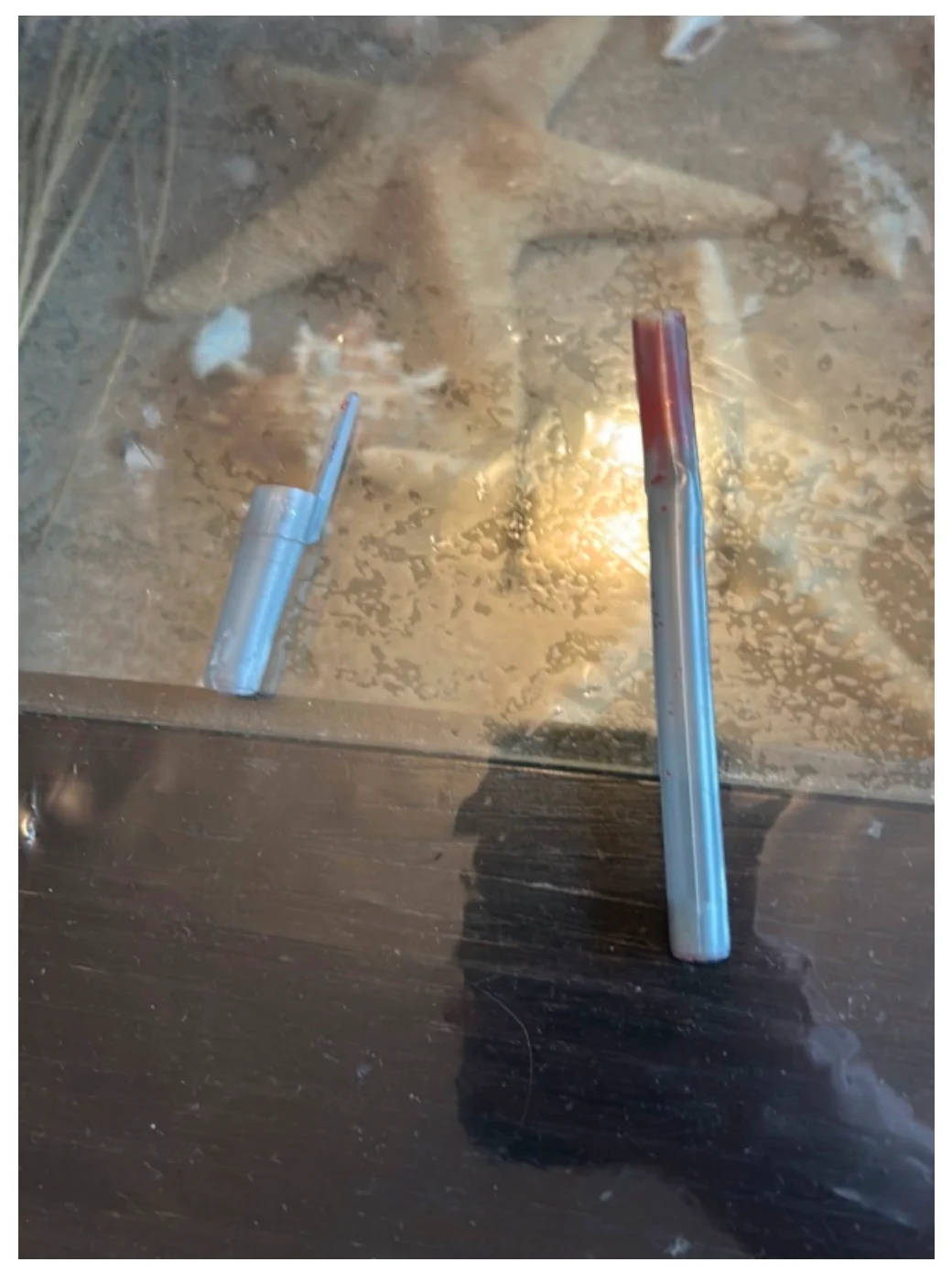
The ballpoint pen found by the patient’s husband.

We present the case of a 46-year-old woman with a history of successfully treated malignancy and no other significant medical comorbidities. Prior to the current presentation, she had exhibited symptoms suggestive of an underlying psychiatric disorder, which had not been recognized or treated. In the days preceding the incident, while on a vacation, several episodes of abnormal behavior indicative of an acute psychiatric disturbance were observed. Following her return home, her husband noticed a marked change in her behavior that was entirely uncharacteristic of her. On the night of the incident, the patient’s husband awoke and discovered that she was no longer in bed. He found her in the living room lying in a pool of blood after apparently inflicting a transnasal self-inflicted injury with a ballpoint pen (Figure 1), with the pen refill missing. Emergency medical services were immediately contacted, and the patient was transported to the University Hospital “St. George”, Plovdiv. Following evaluation in the emergency department by an internist, neurologist, otorhinolaryngologist, psychiatrist, and neurosurgeon, together with a computed tomography (CT) scan of the head, a retained foreign body—a ballpoint pen refill—was identified penetrating through the nasal cavity into the brain parenchyma. The patient was immediately transferred to the operating room for emergency surgical management.

### 2.1. Clinical Examination

The patient was in a stable general condition. She was afebrile, with normal skin and mucosal coloration, clear vesicular breath sounds, and a regular heart rhythm without audible murmurs. Neurological examination revealed no focal neurological deficits. Psychiatric assessment demonstrated a catatonic state with episodes of psychomotor agitation. Local examination showed evidence of spontaneously resolved bleeding from the right nasal cavity. Endoscopic evaluation identified the posterior end of a plastic foreign body superior to the right sphenoid sinus, penetrating through the ethmoidal labyrinth.

### 2.2. Radiological Findings

**Figure 2:**
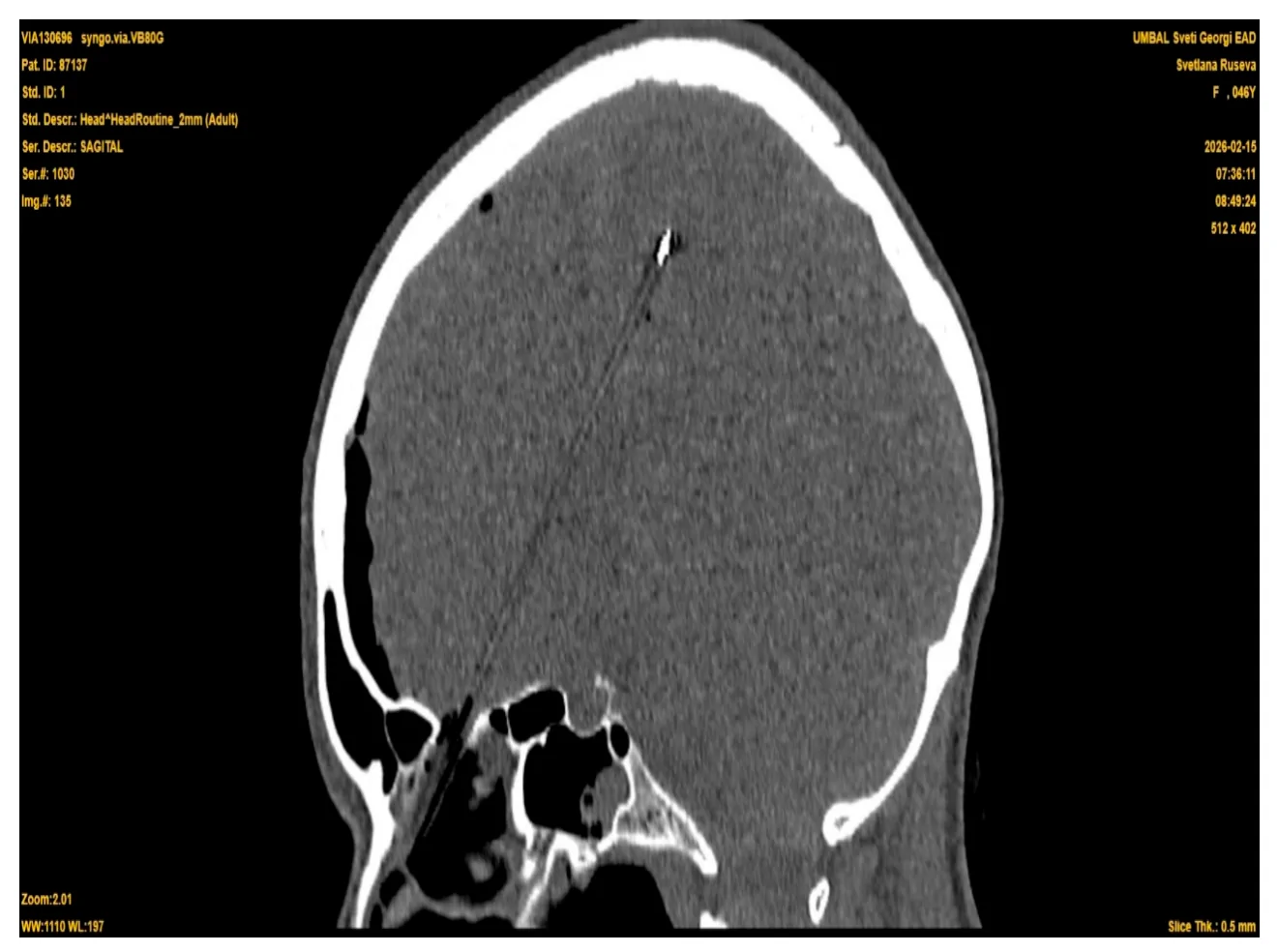
Preoperative CT scans demonstrating the localization of the intracranial foreign body.

**Figure 3:**
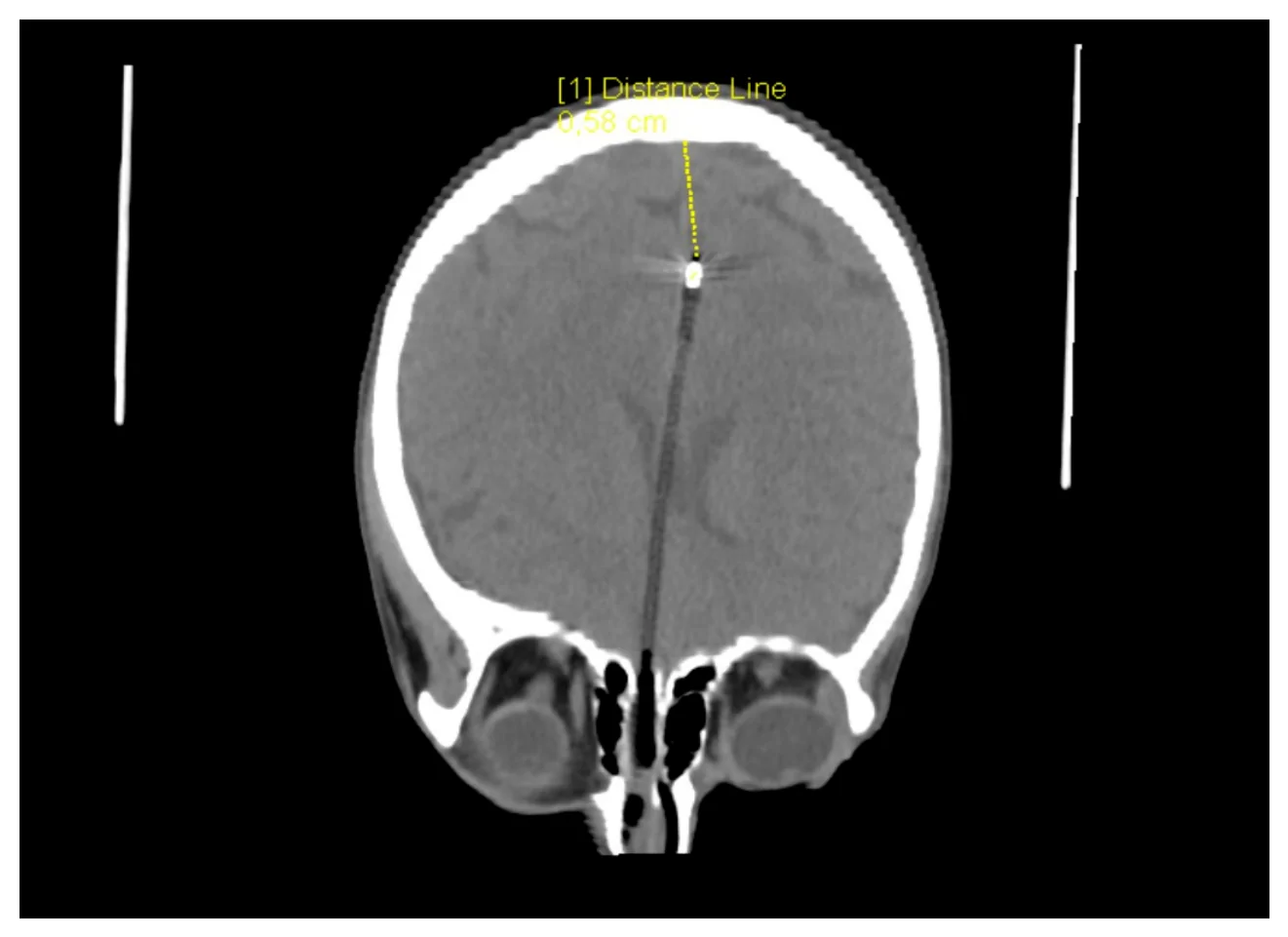
Preoperative CT scans demonstrating the localization of the intracranial foreign body coronal view.

**Figure 4:**
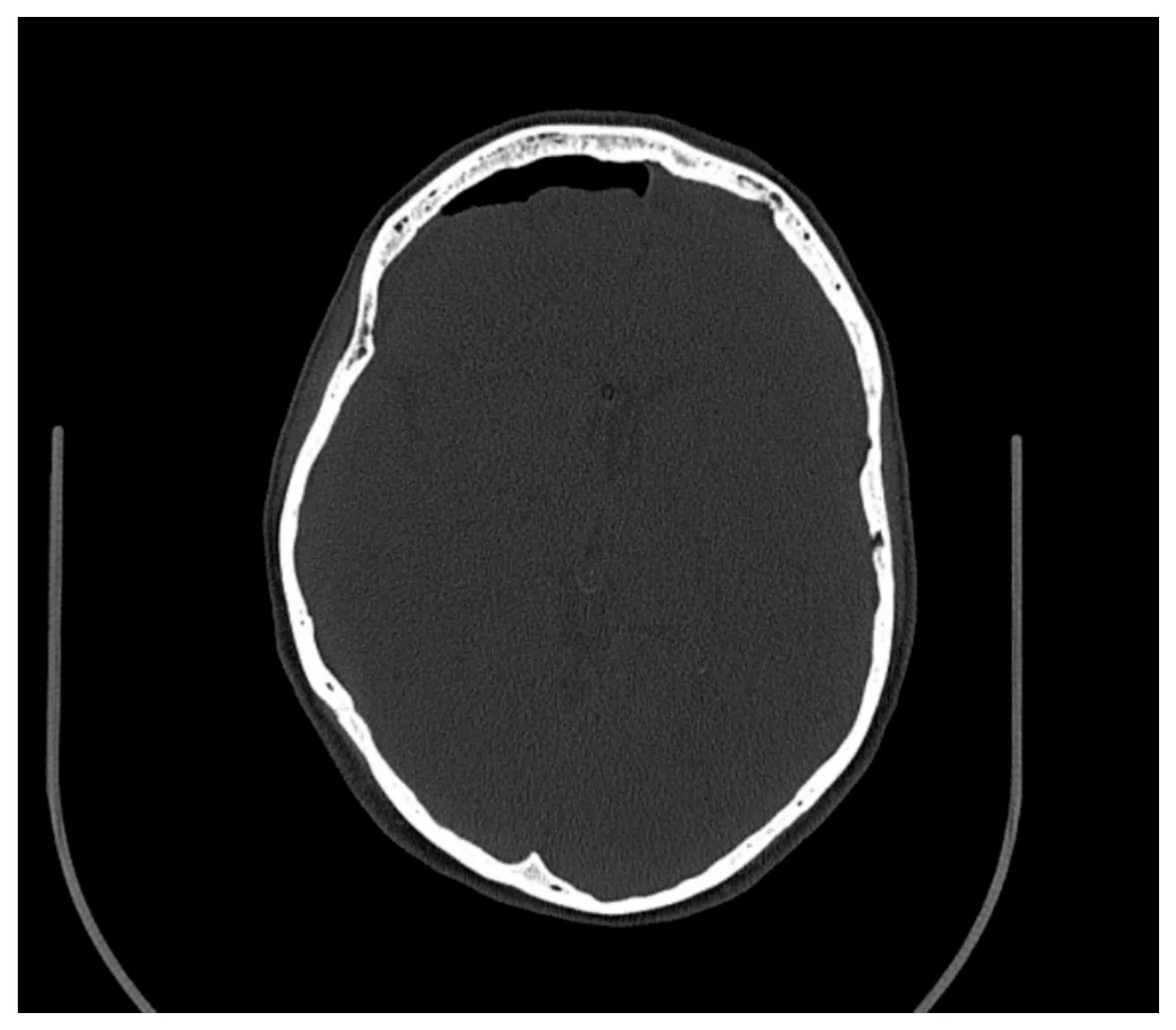
Preoperative CT scan demonstrating intracranial gas collections (pneumocephalus).

Head CT demonstrated a linear hypodense tract extending through the brain parenchyma, traversing the right nasal cavity, the cribriform plate of the ethmoid bone, and the right frontal lobe, reaching the midline near the vertex. At the distal end of the tract, a rounded hyperdense foreign body measuring 5.8 × 6.4 mm was identified (Figure 2 and Figure 3). Intracranial gas collections (pneumocephalus) were present in the right frontotemporal region, with smaller collections in the right frontoparietal region (Figure 4).

### 2.3. Surgical Management

Preoperative assessment and surgical planning were performed by a multidisciplinary team consisting of specialists in vascular surgery, neurosurgery, and otorhinolaryngology (ENT surgery). An endoscopic endonasal removal of the foreign body was planned and performed by an otorhinolaryngologist, with the aim of achieving retrograde extraction along the original trajectory of penetration, thereby minimizing additional injury to the intracranial and cerebral structures. A team of neurosurgeons and vascular surgeons remained on standby throughout the procedure.

**Figure 5:**
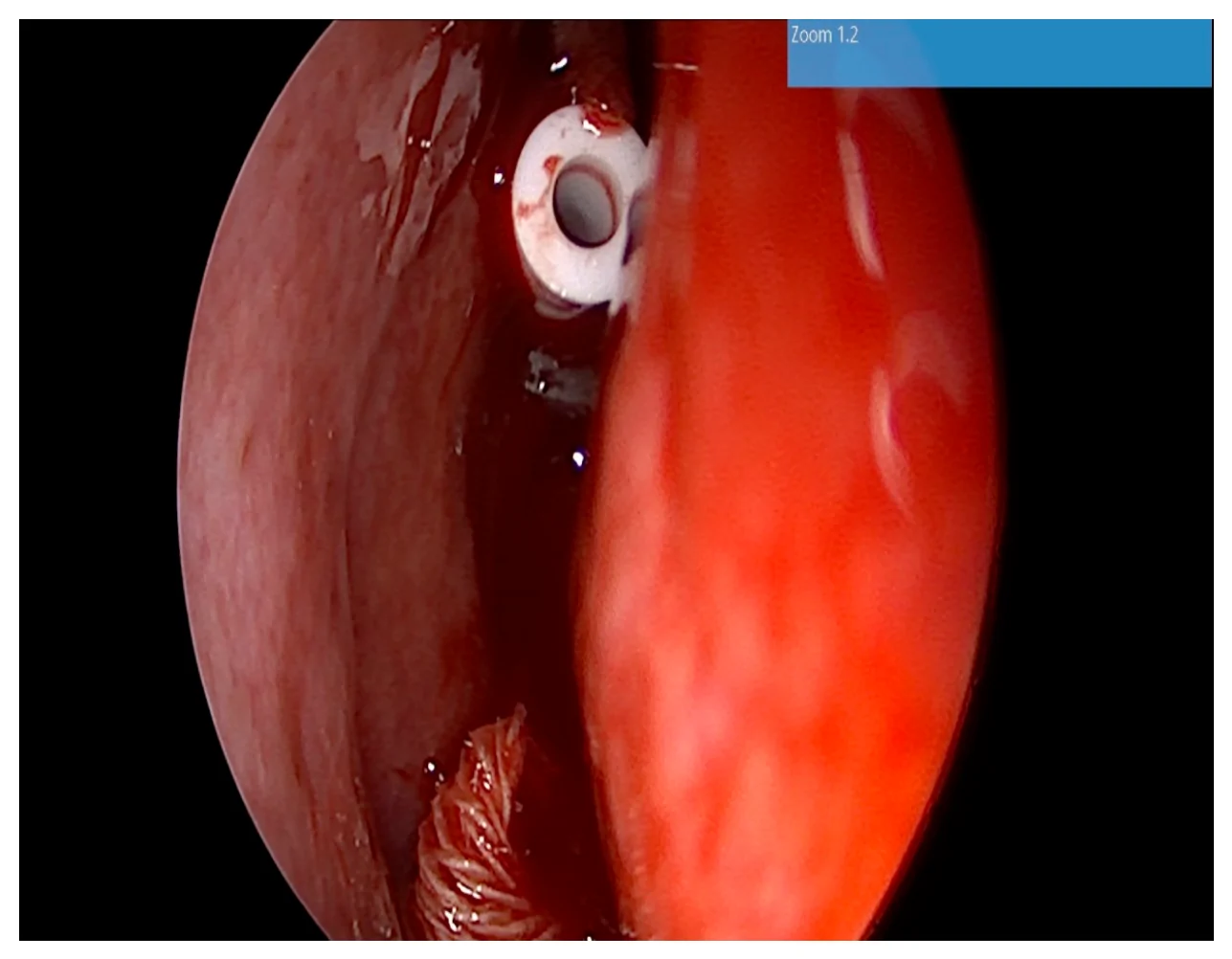
Intraoperative endoscopic localization of the foreign body.

**Figure 6:**
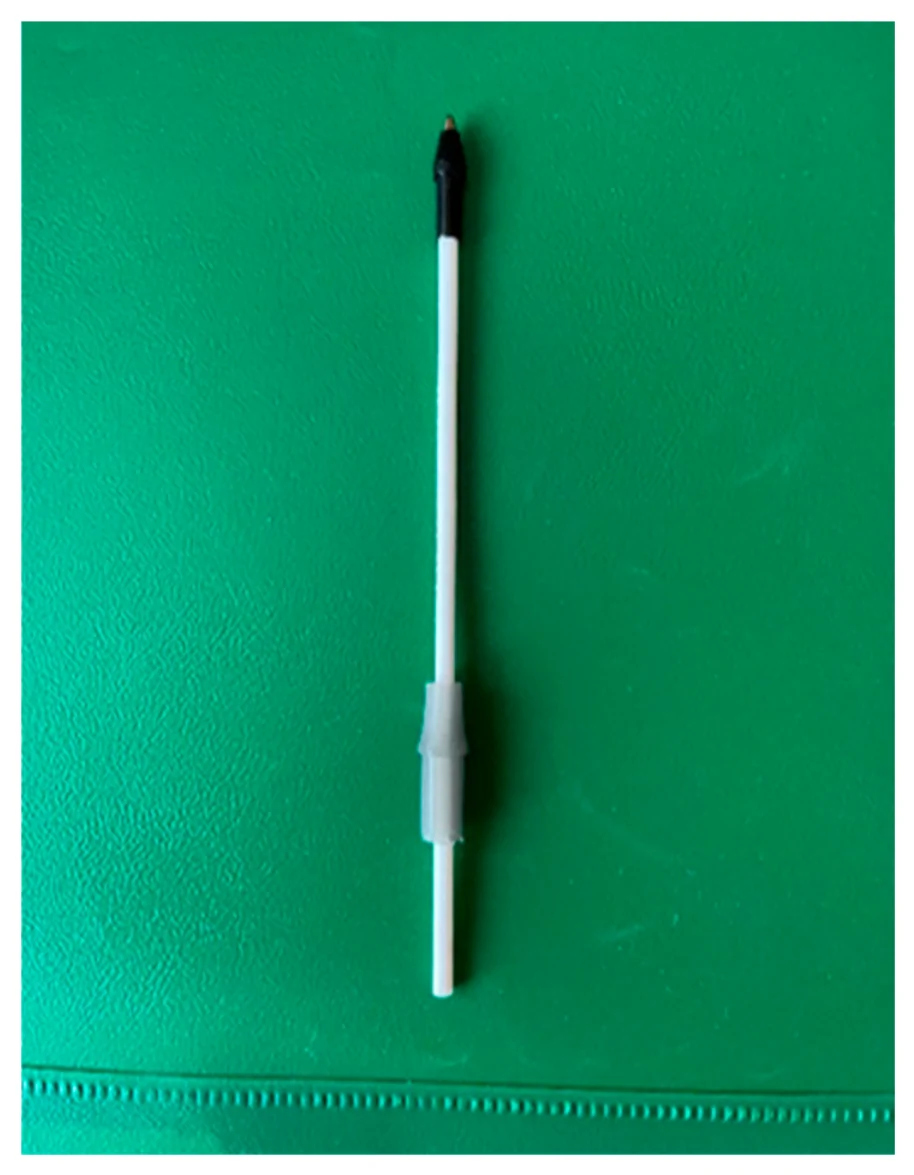
The extracted foreign body (ballpoint pen refill).

Under general anesthesia, following removal of the vasoconstrictor-soaked nasal packs, the right nasal cavity was examined using a 0° rigid endoscope. The proximal end of the foreign body (a ballpoint pen refill) was identified superior to the right sphenoid sinus (Figure 5). The right middle turbinate was resected, followed by stepwise anterior, middle, and posterior ethmoidectomy to expose the skull base (anterior skull base) and identify the site of penetration through the cribriform plate. After complete exposure of the skull base defect, the foreign body was gently removed along its original trajectory without resistance (Figure 6).

Following removal of the ballpoint pen refill, intraoperative inspection revealed a skull base defect measuring approximately 4 × 5 mm, located immediately superior to the right sphenoid sinus without extension into the sinus cavity.

**Figure 7:**
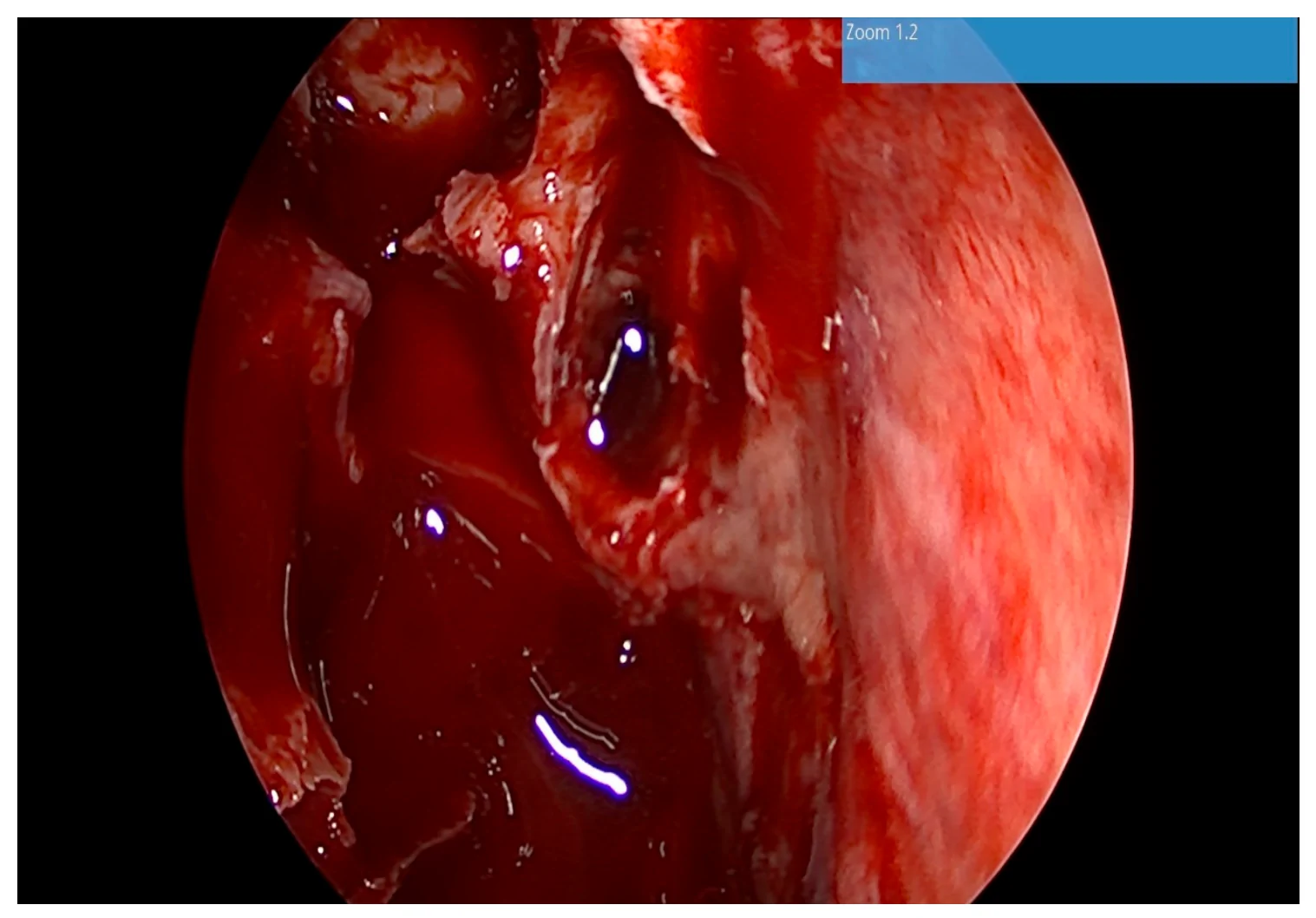
Skull base defect at the anterior skull base with cerebrospinal fluid (CSF) leak following removal of the foreign body.

**Figure 8:**
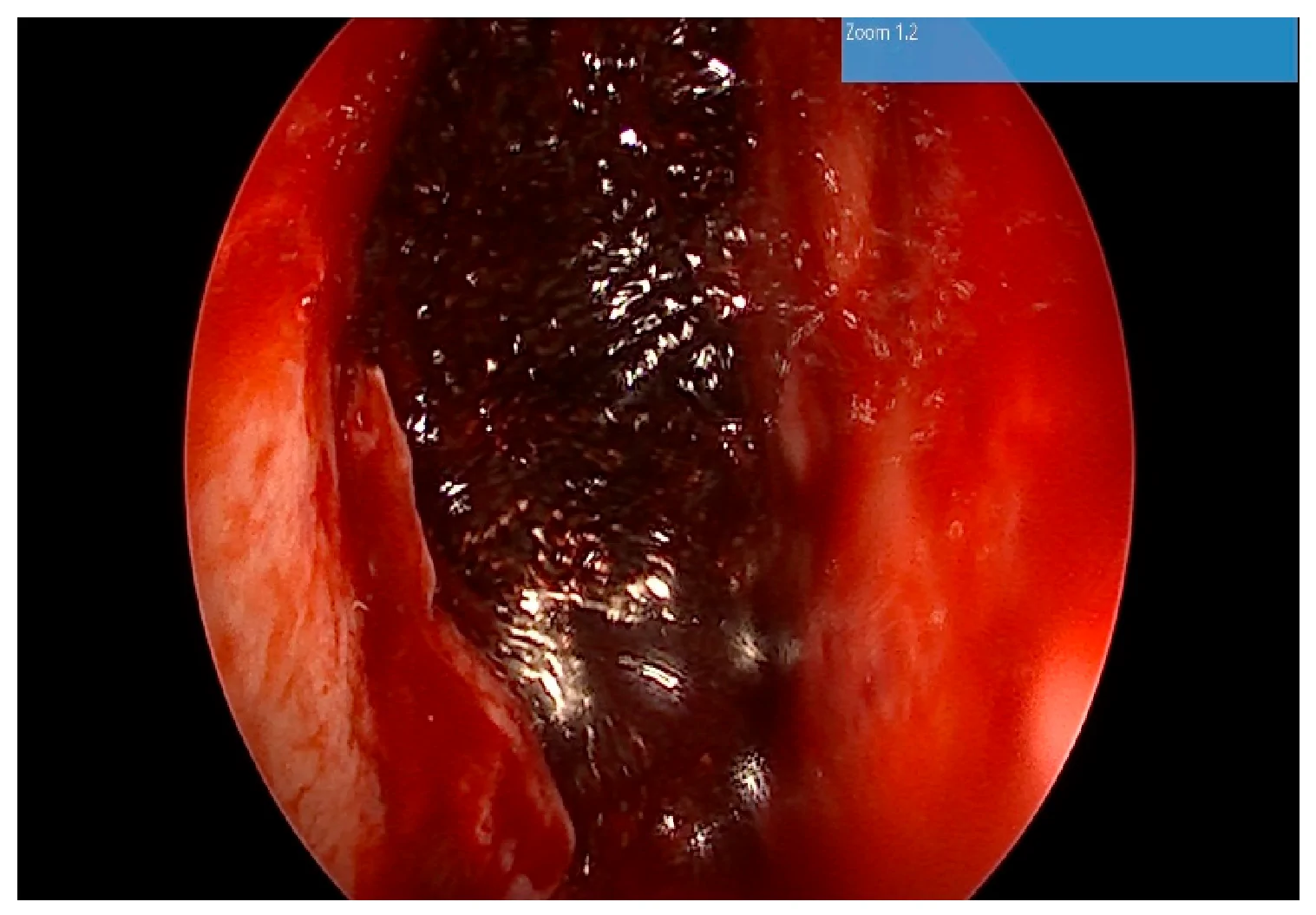
Reconstruction of the skull base (anterior skull base) defect with oxidized regenerated cellulose (Surgicel^®^).

**Figure 9:**
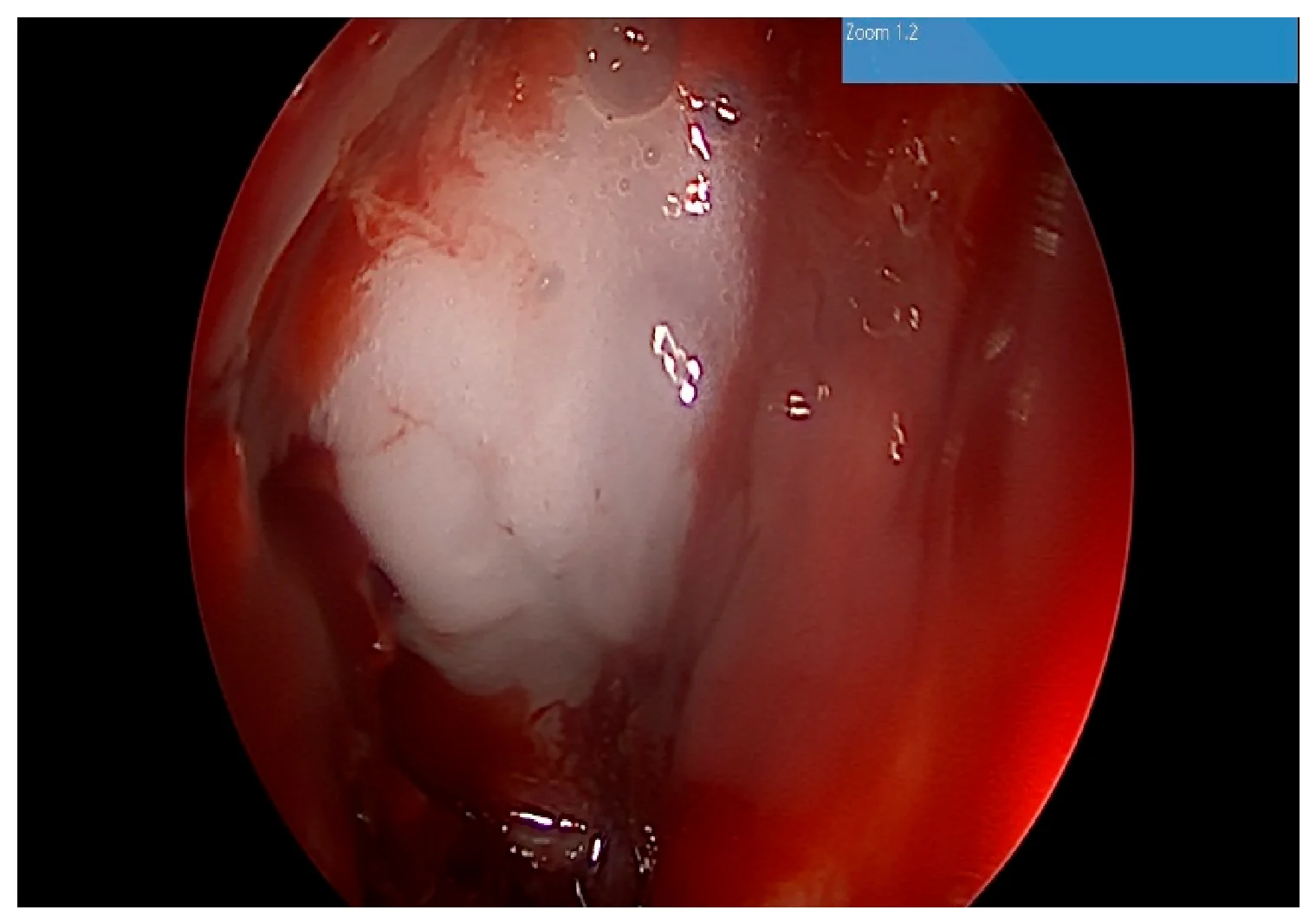
Reconstruction of the skull base (anterior skull base) defect with tissue adhesive.

Following removal of the foreign body, cerebrospinal fluid (CSF) leakage was identified through the traumatic defect of the anterior skull base and the dura mater (Figure 7). To achieve a watertight closure, the skull base defect was reconstructed using a multilayer technique with an autologous fascia lata graft harvested from the patient’s thigh. The reconstruction was further reinforced with oxidized regenerated cellulose (Surgicel^®^, Johnson & Johnson Medical GmbH, Norderstedt, Germany) (Figure 8) and tissue adhesive (Figure 9). A gelatin sponge (Gelaspon^®^, Baxter Healthcare SA, Zurich, Switzerland) was placed over the reconstruction, and the right nasal cavity was packed with three glove-finger nasal packs.

Postoperatively, the patient received broad-spectrum intravenous antibiotic therapy according to the institutional protocol for penetrating intracranial injuries to reduce the risk of intracranial and sinonasal infectious complications.

**Figure 10:**
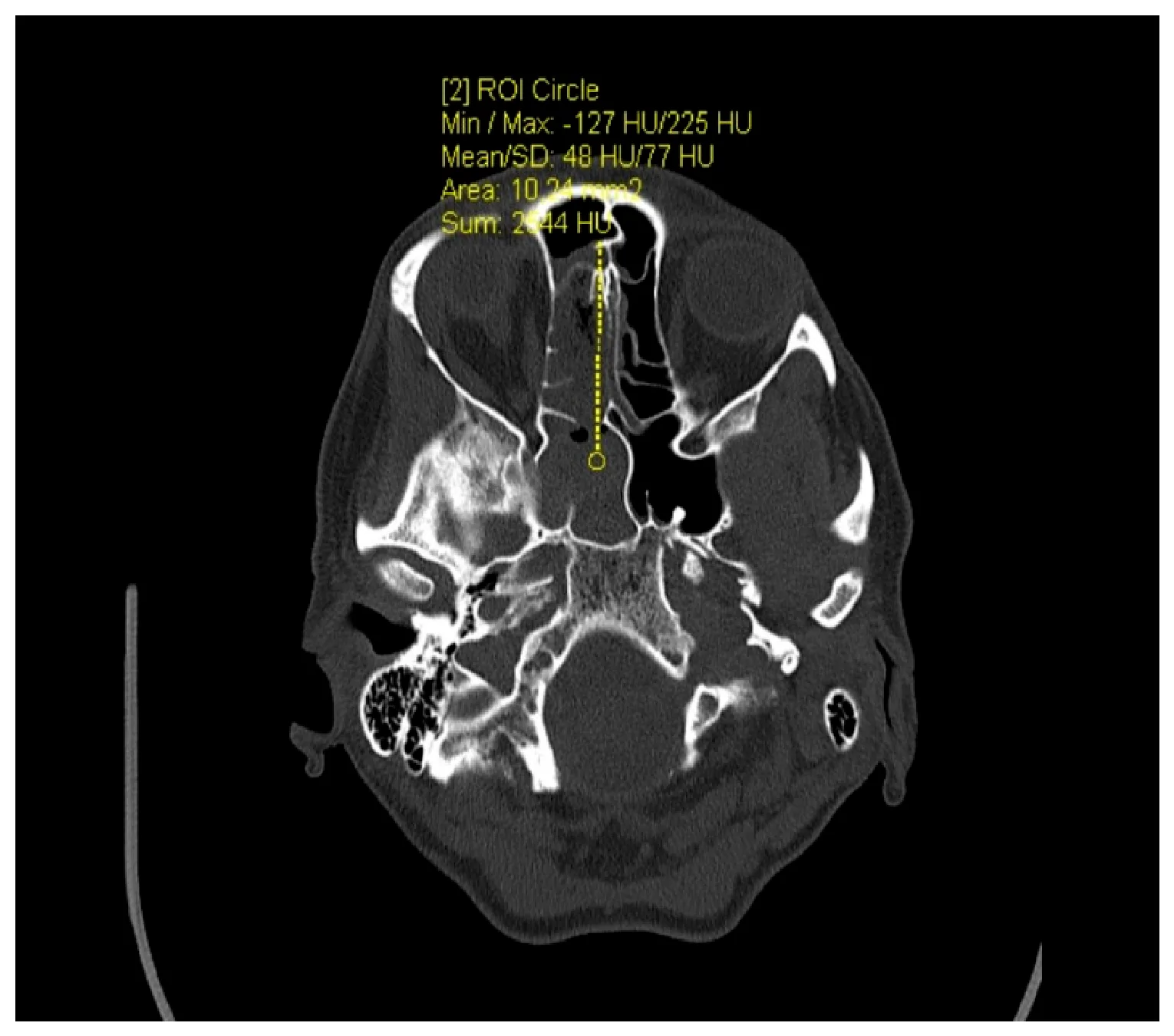
Postoperative changes along the trajectory of the foreign body with persistent pneumocephalus, sagittal view.

**Figure 11:**
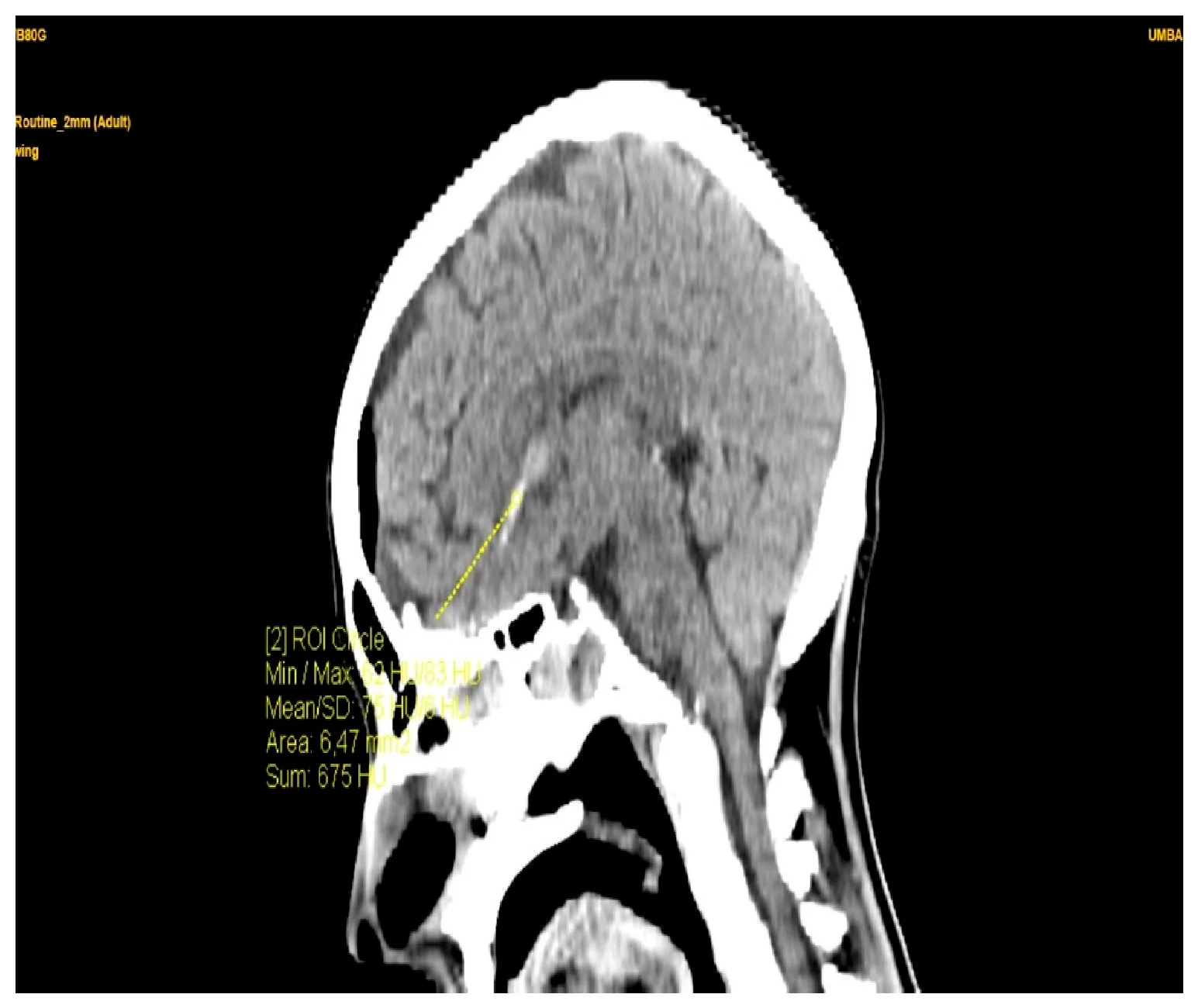
Postoperative changes along the trajectory of the foreign body with persistent pneumocephalus.

The postoperative course was uneventful. The patient was monitored in the neurosurgical department by a neurosurgeon, an otorhinolaryngologist, and a psychiatrist. Nasal packing was removed on the 48th postoperative hour. A follow-up CT scan was performed, demonstrating a traumatic skull base defect with small hemorrhagic foci along the trajectory of the foreign body, with persistence of pneumocephalus (Figure 10 and Figure 11).

**Figure 12:**
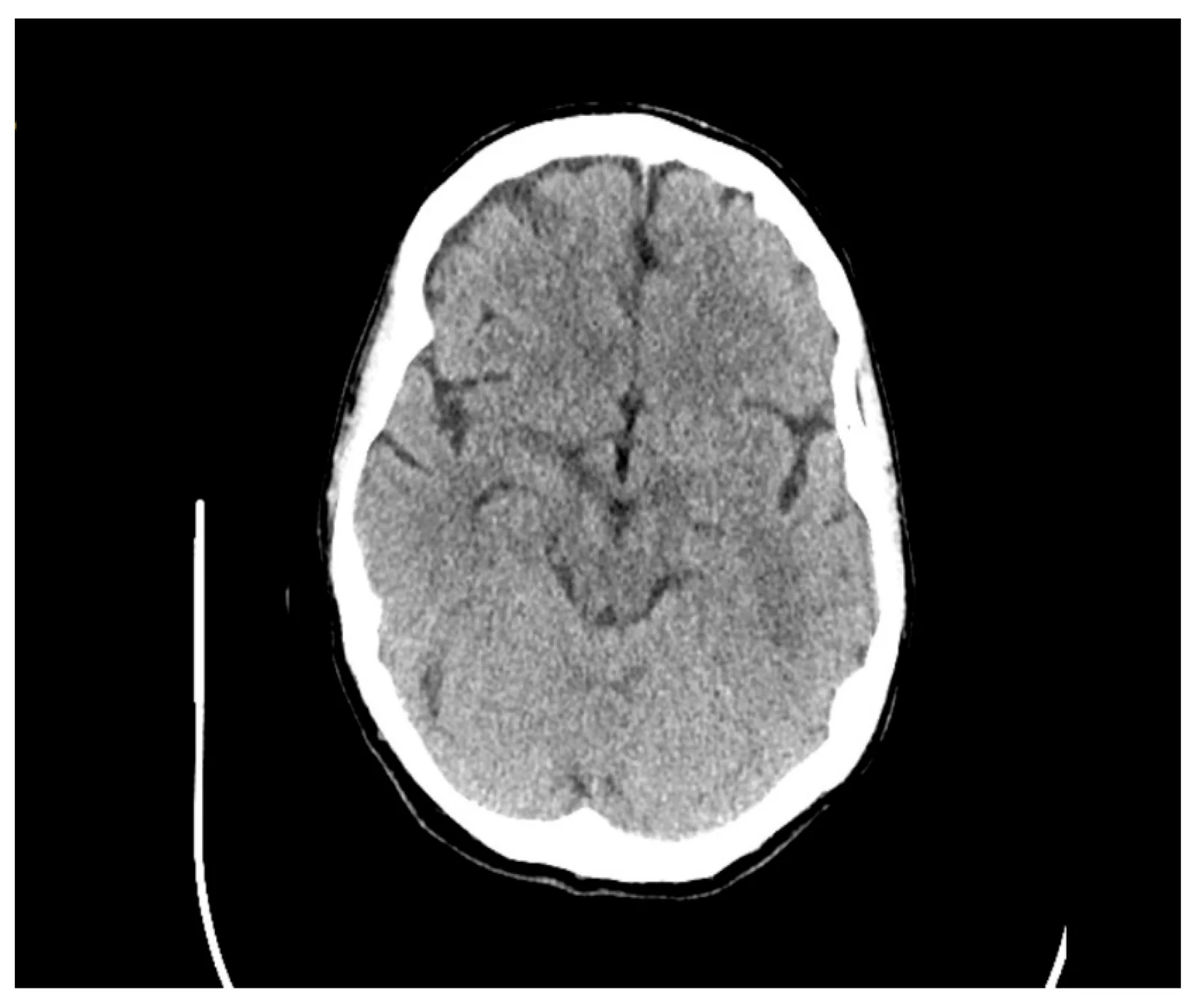
Follow-up CT images demonstrating progressive resolution of the intracerebral hemorrhages and pneumocephalus.

**Figure 13:**
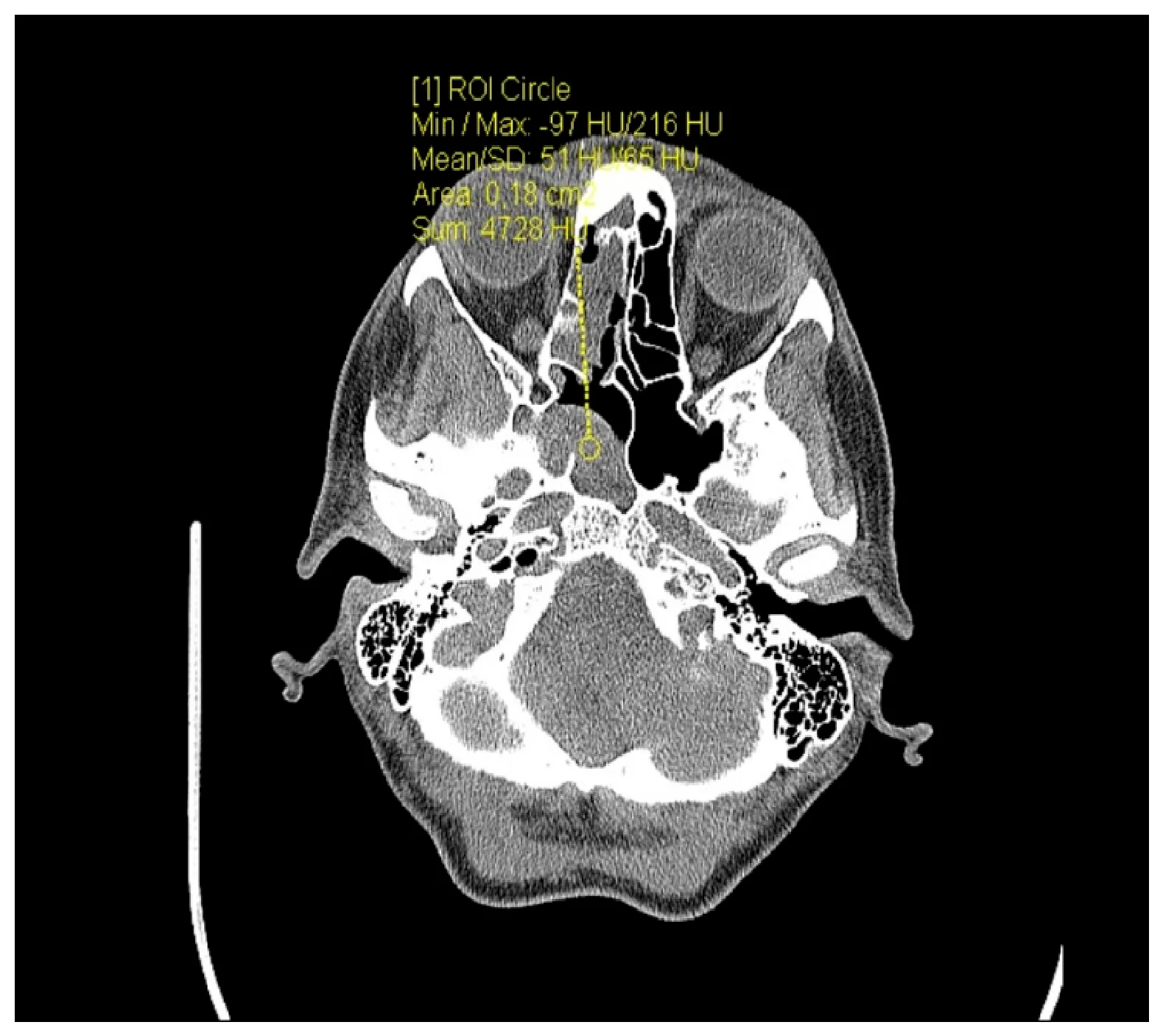
Follow-up CT images demonstrating progressive resolution of the intracerebral hemorrhages and pneumocephalus.

Nine days later, follow-up CT imaging demonstrated near-complete regression of the hemorrhagic collections and complete resolution of the pneumocephalus (Figure 12 and Figure 13). The patient was discharged on the 25th postoperative day in an afebrile condition, fully oriented, without headache or rhinorrhea, and was referred to the Department of Psychiatry for further management. The psychiatric diagnosis was established as an acute polymorphic psychotic disorder with schizophrenic symptoms. After an additional 25 days of inpatient psychiatric treatment, the patient was discharged in good general condition with maintenance therapy prescribed for outpatient follow-up.

## 3. Discussion

Transnasal penetrating brain injuries are exceptionally rare, accounting for only a small proportion of penetrating craniofacial trauma [1]. Most penetrating craniofacial injuries occur through the temporal bone or the orbit [10], whereas transnasal injuries are reported predominantly in children following accidental trauma during play. Suicide attempts involving self-inflicted penetrating craniofacial injuries have been reported predominantly in male patients [7]. Deliberate transnasal intracranial penetration through the nasal cavity therefore represents an exceptionally uncommon clinical entity.

To better contextualize the present case, the principal characteristics of previously reported transnasal penetrating intracranial injuries are summarized in Table 1. It clearly demonstrates the wide variety of objects responsible for this type of injury. Notably, among the cases reported between 2000 and 2026, only three were caused by a ballpoint pen [11,12,13].

The main epidemiological and clinical trends identified from the published literature are presented in Table 2.

The present case exhibits several distinctive features. First, unlike the few previously reported cases of transnasal penetrating brain injury, including those caused by umbrella tips, no external lacerations or visible injuries of the nostril were identified on clinical examination. Second, the foreign body remained deeply impacted within the ethmoidal labyrinth and the right frontal lobe. In contrast to previously reported cases, in which the penetrating object had either been withdrawn or had exited the cranial cavity before presentation, the retained ballpoint pen refill remained in situ along its entire intracranial trajectory, allowing accurate radiological localization, meticulous preoperative planning, and controlled endoscopic endonasal removal.

The clinical outcome of penetrating intracranial injuries depends on several factors, including the site of injury, the depth of penetration, and the intracranial structures traversed by the foreign body. Prognosis is further influenced by secondary complications, such as intracranial hematoma formation and traumatic pneumocephalus. According to the available literature, a considerable proportion of patients remain conscious following the traumatic event, as observed in our patient. The neurological outcome is determined primarily by the location and extent of damage to critical intracranial structures.

In the present case, the ballpoint pen refill entered through the right nasal cavity and penetrated the cranial cavity superior to the right sphenoid sinus, closely following the anatomical corridor commonly used for endoscopic transsphenoidal pituitary surgery. Remarkably, the foreign body advanced intracranially without injuring the cavernous sinus or the internal carotid artery and ultimately penetrated the right frontal lobe without involving eloquent cortical areas or other critical neurovascular structures. This favorable anatomical trajectory most likely explains the complete absence of neurological deficits.

In contrast, previously reported cases have frequently been associated with neurological impairment, with recovery often being slow or incomplete. Penetrating injuries involving the internal carotid artery or the cavernous sinus are associated with a substantially poorer prognosis and are frequently fatal because of catastrophic intracranial hemorrhage.

Although the reported mortality associated with penetrating intracranial injuries remains high (approximately 50%) [3], the prognosis is generally more favorable than traumatic brain injuries caused by high-velocity projectiles [14]. In the absence of catastrophic hemorrhage resulting from injury to major intracranial vessels, the greatest risk associated with transnasal skull base penetration is the development of severe intracranial infections, particularly bacterial meningitis, which may be life-threatening.

Additional complications may also arise during surgical removal of the retained foreign body. According to published data, the penetrating object has already been removed from the wound tract before hospital admission in more than 76% of patients [15], making accurate assessment of the trajectory, identification of associated structural damage, and appropriate surgical planning considerably more challenging.

## 4. Conclusions

Transnasal penetrating intracranial injuries are exceptionally rare but potentially life-threatening conditions that require prompt diagnosis and meticulous multidisciplinary management. The present case demonstrates that a retained intracranial foreign body can be successfully treated using an endoscopic endonasal approach when its anatomical trajectory is carefully evaluated with high-resolution imaging and appropriate surgical planning.

Comprehensive preoperative radiological assessment is essential for defining the trajectory of the foreign body, identifying associated intracranial injuries, and excluding major neurovascular involvement. Endoscopic endonasal removal, combined with definitive anterior skull base reconstruction, enables controlled retrograde extraction while minimizing additional tissue injury and effectively managing cerebrospinal fluid leakage.

Successful treatment also depends on the prevention of infectious complications through appropriate perioperative management and close postoperative follow-up. This case highlights the safety and effectiveness of a multidisciplinary approach involving otorhinolaryngologists, neurosurgeons, radiologists, anesthesiologists, and psychiatrists in achieving favorable clinical outcomes in carefully selected patients with transnasal penetrating intracranial injuries.

## Figures and Tables

**Table 1:** Some Published Cases of Transnasal Penetrating Intracranial Injuries Reported in the Literature.

Reference	Patient	Foreign Body	Mechanism	Surgical Management	Outcome
Ihama et al. [1]	Adult	Plastic-covered umbrella tip	Accidental	Surgical removal	Recovery
Yamamoto et al. [2]	Child	Chopstick	Accidental	Craniotomy	Recovery
Fallon et al. [3]	Child	Wooden stick	Accidental	Surgical removal	Recovery
Nishio et al. [5]	Child	Wooden chopstick	Accidental	Craniotomy and abscess drainage	Recovery
Kaiser et al. [6]	Child	Pencil	Accidental	Surgical removal	Recovery
Yilmaz et al. [8]	Child	Sewing needle	Infanticide attempt	Surgical removal	Recovery
Yoneoka et al. [9]	Adult	Garden pole	Accidental	Endoscopic endonasal skull base reconstruction	Recovery
Ha Son Nguyen et al. [11]	Adult	Ballpoint pen	Self-inflicted	Endovascular embolization followed by combined transnasal endoscopy and open craniotomy for pen removal	Recovery
Philippe Lunetta et al. [12]	Adult	ballpoint pen	Self-inflicted	Misdiagnosed	Died
Chi-Man Yip [13]	Adult	ballpoint pen	Self-inflicted	Surgical removal	Recovery
Present case	46/F	Ballpoint pen refill	Self-inflicted	Endoscopic endonasal removal with anterior skull base reconstruction	Complete neurological recovery

**Table 2:** Summary of the Published Literature on Transnasal Penetrating Intracranial Injuries.

Characteristic	Summary
Predominant age group	Pediatric patients
Adult patients	Uncommon
Most frequent mechanism	Accidental trauma
Self-inflicted injuries	Exceptionally rare; predominantly reported in males [7]
Most common foreign bodies	Wooden objects (chopsticks, sticks), pencils, sewing needles, umbrella tips
Retained foreign body at presentation	Uncommon; in most cases the object had been removed before hospital admission [11]
Common intracranial complications	Pneumocephalus, intracranial hemorrhage, cerebrospinal fluid leakage, meningitis, brain abscess
Major prognostic factors	Injury to the internal carotid artery, cavernous sinus, or eloquent brain regions; delayed diagnosis; intracranial infection

## Data Availability

The data are available from the author upon reasonable request.

## References

[1] Ihama Y., Nagai T., Ninomiya K., Fukasawa M., Fuke C., Miyazaki T. (2012). A transnasal intracranial stab wound by a plastic-covered umbrella tip. Forensic Sci. Int..

[2] Yamamoto I., Yamada S., Sato O. (1985). Unusual craniocerebral penetrating injury by a chopstick. Surg. Neurol..

[3] Fallon M.J., Plante D.M., Brown L.W. (1992). Wooden transnasal intracranial penetration: An unusual presentation. J. Emerg. Med..

[4] Holmgren E., Schartz D., Puttige Ramesh N., Sylvester K., Eskey C. (2020). Penetrating midface trauma: A case report, review of the literature, and a diagnostic and management protocol. J. Oral Maxillofac. Surg..

[5] Nishio Y., Hayashi N., Hamada H., Hirashima Y., Endo S. (2004). A case of delayed brain abscess due to a retained intracranial wooden foreign body: A case report and review of the last 20 years. Acta Neurochir..

[6] Kaiser M.C., Rodesch G., Capesius P. (1983). CT in a case of intracranial penetration of a pencil. Neuroradiology.

[7] Oden C., Weinschreider E., Linzie H. (2023). Self-inflicted stabbings as nonfatal suicide attempts: A systematic review and case series. J. Acad. Consult. Liaison Psychiatry.

[8] Yilmaz N., Kiymaz N., Yilmaz C., Bay A., Mumcu C. (2007). Intracranial foreign bodies causing delayed brain abscesses: Intracranial sewing needles. J. Neurosurg. Pediatr..

[9] Yoneoka Y., Aizawa N., Nonomura Y., Ogi M., Seki Y., Akiyama K. (2020). Traumatic nonmissile penetrating transnasal anterior skull base fracture and brain injury with cerebrospinal fluid leak: Intraoperative leak detection and an effective reconstruction procedure for a localized skull base defect especially after coronavirus disease 2019 outbreak. World Neurosurg..

[10] Dempsey L.C., Winestock D.P., Hoff J.T. (1977). Stab wounds of the brain. West. J. Med..

[11] Nguyen H.S., Oni-Orisan A., Doan N., Mueller W. (2016). Transnasal Penetration of a Ballpoint Pen: Case Report and Review of Literature. World Neurosurg..

[12] Lunetta P., Ohberg A., Sajantila A. (2002). Suicide by intracerebellar ballpoint pen. Am. J. Forensic Med. Pathol..

[13] Yip C.-M. (2012). Attempt Suicide by Inserting a Ball-Pen into the Brain: A Case Report. Surg. Sci..

[14] Cardicott D.G., Pearce A., Price R., Croser D., Brophy B. (2004). Not just another ‘head lac’ low-velocity, penetrating intro-cranial injuries: A case report and review of the literature. Inj. Int. J. Care Inj..

[15] Trevou M.D., Dellen J.R. (1992). Penetrating stab wounds to the brain: The timing of angiography in patients presenting with the weapon already removed. Neurosurgery.

